# Late Na^+^ Current Is [Ca^2+^]_i_-Dependent in Canine Ventricular Myocytes

**DOI:** 10.3390/ph14111142

**Published:** 2021-11-11

**Authors:** Dénes Kiss, Balázs Horváth, Tamás Hézső, Csaba Dienes, Zsigmond Kovács, Leila Topal, Norbert Szentandrássy, János Almássy, János Prorok, László Virág, Tamás Bányász, András Varró, Péter P. Nánási, János Magyar

**Affiliations:** 1 Department of Physiology, Faculty of Medicine, University of Debrecen, 4032 Debrecen, Hungary; kiss.denes@med.unideb.hu (D.K.); horvath.balazs@med.unideb.hu (B.H.); hezso.tamas@med.unideb.hu (T.H.); dienes.csaba@med.unideb.hu (C.D.); kovacs.zsigmond@med.unideb.hu (Z.K.); szentandrassy.norbert@med.unideb.hu (N.S.); almassy.janos@med.unideb.hu (J.A.); banyasz.tamas@med.unideb.hu (T.B.); magyar.janos@med.unideb.hu (J.M.); 2 Faculty of Pharmacy, University of Debrecen, 4032 Debrecen, Hungary; 3 Department of Pharmacology and Pharmacotherapy, Faculty of Medicine, University of Szeged, 6725 Szeged, Hungary; topal.leila@gmail.com (L.T.); prorok.janos@med.u-szeged.hu (J.P.); virag.laszlo@med.u-szeged.hu (L.V.); varro.andras@med.u-szeged.hu (A.V.); 4 Department of Basic Medical Sciences, Faculty of Dentistry, University of Debrecen, 4032 Debrecen, Hungary; 5 ELKH-SZTE Research Group for Cardiovascular Pharmacology, Eötvös Loránd Research Network, 6725 Szeged, Hungary; 6 Department of Pharmacology and Pharmacotherapy, Interdisciplinary Excellence Centre, University of Szeged, 6725 Szeged, Hungary; 7 Department of Dental Physiology and Pharmacology, Faculty of Dentistry, University of Debrecen, 4032 Debrecen, Hungary; 8 Division of Sport Physiology, Department of Physiology, Faculty of Medicine, University of Debrecen, 4032 Debrecen, Hungary

**Keywords:** late Na^+^ current, cytosolic Ca^2+^ concentration, CaMKII, action potential voltage clamp, canine myocytes

## Abstract

Enhancement of the late sodium current (I_NaL_) increases arrhythmia propensity in the heart, whereas suppression of the current is antiarrhythmic. In the present study, we investigated I_NaL_ in canine ventricular cardiomyocytes under action potential voltage-clamp conditions using the selective Na^+^ channel inhibitors GS967 and tetrodotoxin. Both 1 µM GS967 and 10 µM tetrodotoxin dissected largely similar inward currents. The amplitude and integral of the GS967-sensitive current was significantly smaller after the reduction of intracellular Ca^2+^ concentration ([Ca^2+^]_i_) either by superfusion of the cells with 1 µM nisoldipine or by intracellular application of 10 mM BAPTA. Inhibiting calcium/calmodulin-dependent protein kinase II (CaMKII) by KN-93 or the autocamtide-2-related inhibitor peptide similarly reduced the amplitude and integral of I_NaL_. Action potential duration was shortened in a reverse rate-dependent manner and the plateau potential was depressed by GS967. This GS967-induced depression of plateau was reduced by pretreatment of the cells with BAPTA-AM. We conclude that (1) I_NaL_ depends on the magnitude of [Ca^2+^]_i_ in canine ventricular cells, (2) this [Ca^2+^]_i_-dependence of I_NaL_ is mediated by the Ca^2+^-dependent activation of CaMKII, and (3) I_NaL_ is augmented by the baseline CaMKII activity.

## 1. Introduction

Following the large sodium current surge associated with the upstroke of the non-pacemaker action potential (AP) in the heart, a smaller but sustained current component called late sodium current (I_NaL_) remains active throughout the entire AP. I_NaL_ contributes to plateau formation and is responsible for largely half of the transmembrane Na^+^ entry through voltage-dependent Na^+^ channels [1,2,3]. Native I_NaL_ is underlain by several different mechanisms, including the overlap between steady-state voltage-dependent activation and inactivation of the Na^+^ channels (window Na^+^ current) [4], burst mode and late scattered mode channel openings [5,6], non-equilibrium channel gating [7], and non-cardiac sodium channel isoforms [8].

I_NaL_ has a well-established physiological significance [9,10,11], and an important pathophysiological role in heart failure and LQT3 syndrome [12,13,14]. An increased I_NaL_ leads to higher arrhythmia propensity because of prolongation of the action potential duration (APD), increased inhomogeneity of repolarization and occurrence of afterdepolarizations. On the other hand, suppression of the current is antiarrhythmic in a variety of animal models [12,15,16,17]. Despite its pathophysiological importance, many aspects of I_NaL_ are still poorly understood. One of these is the physiological regulation of the current, which seems to be a target of phosphorylation by protein kinase A, protein kinase C, as well as calcium/calmodulin-dependent protein kinase II (CaMKII) [18,19]. Indeed, CaMKII was shown to increase the amplitude of I_NaL_ providing a pathway for [Ca^2+^]_i_-dependent augmentation of the current in rabbit [18,19,20,21], murine [21,22,23], guinea pig [24] and porcine [25] cardiomyocytes.

Under action potential voltage clamp (APVC) conditions, canine myocytes are considered a reasonably good model for human ventricular cells regarding many ionic currents [26,27,28,29]. Our recent study also shows that the shape of I_NaL_ under the AP is quite similar in dogs and humans [30], while being quite different from I_NaL_ in other mammals, like guinea pigs [24], rabbits [18] and pigs [25]. Despite the similarity between canine and human I_NaL_, only a limited number of studies were conducted on canine myocytes [13,14,31]. Additionally, most of the available data were obtained under conditions far from being physiological, using conventional voltage clamp arrangements at room temperature with intracellular Ca^2+^ buffering. In the present study, we used an experimental approach that is much closer to in vivo physiological conditions. We measured I_NaL_ under a ventricular action potential with APVC using the selective I_NaL_ inhibitor GS967 (mentioned also as GS-458967). We also performed our experiments at 37 °C, with normal intracellular Ca^2+^ homeostasis [32,33,34]. We found that I_NaL_ is Ca^2+^-dependent, and the baseline calcium/calmodulin-dependent protein kinase II (CaMKII) activity augments I_NaL_ in single canine ventricular myocytes. These results significantly improve our understanding of physiological regulation of the cardiac action potential and mechanism towards pathological conditions.

## 2. Results

### 2.1. Effects of GS967 and TTX on I_NaL_ under Action Potential Voltage Clamp Conditions

**Figure 1:**
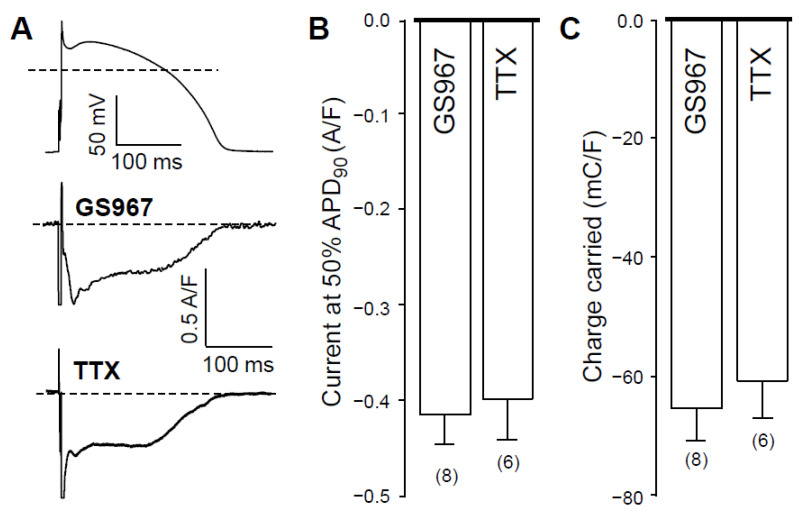
Effects of GS967 and TTX on I_NaL_ in isolated canine ventricular myocytes under action potential voltage-clamp conditions. (**A**) representative membrane current records dissected by 1 µM GS967 and 10 µM TTX in Tyrode solution. The command AP is shown above current traces. Dashed lines indicate zero voltage and current levels. (**B**) Current densities measured at 50% of APD_90_. (**C**) Current integrals (charge carried) from which the initial 20 ms period were excluded. Columns and bars denote mean ± SEM values, numbers in parentheses indicate the number of myocytes studied.

**Figure 2:**
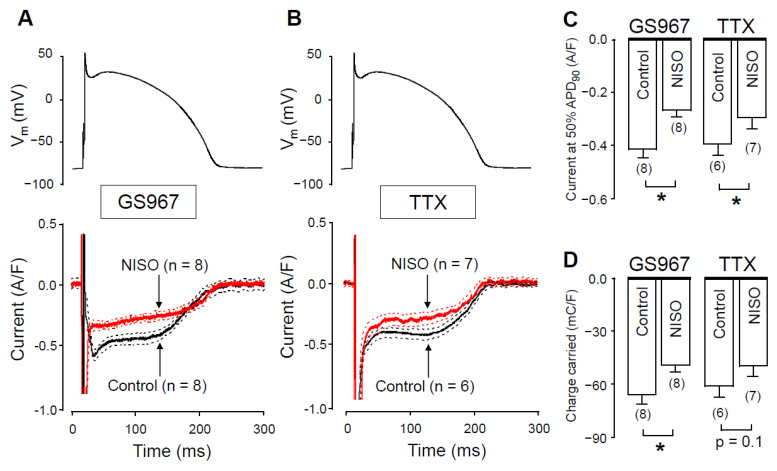
GS967-sensitive and TTX-sensitive currents were recorded under action potential voltage-clamp conditions in the absence and presence of nisoldipine. (**A**,**B**) The command AP (above), GS967-sensitive (**A**) and TTX-sensitive (**B**) current profiles (below) obtained in the presence (NISO) and absence (Control) of 1 µM nisoldipine. Dashed lines denote SEM values. (**C**,**D**) Average current densities, measured at 50% of APD_90_ (**C**), and current integrals (**D**) obtained in the absence and presence of nisoldipine with GS967 and TTX. Columns and bars are mean ± SEM, numbers in parentheses indicate the number of myocytes studied, asterisks denote significant differences between the NISO and control groups.

Under action potential voltage-clamp conditions, 1 µM GS967 and 10 µM TTX dissected similar inward current profiles in canine ventricular cells (Figure 1A). In cardiac myocytes, both agents selectively inhibit Na^+^ currents. These concentrations were chosen because the densities, measured at 50% of APD_90_ (−0.42 ± 0.03 versus −0.40 ± 0.04 A/F) and integrals (−68 ± 5 versus −61 ± 6 mC/F) of the dissected currents were largely comparable in size (see Figure 1B,C, respectively). We studied the Ca^2+^-sensitivity of I_NaL_ by blocking L-type calcium current (I_Ca_) with 1 µM nisoldipine (NISO) in order to reduce the Ca^2+^ entry into the myocytes. As demonstrated in Figure 2, I_NaL_ was smaller following nisoldipine pretreatment than under control conditions. Both in the case of GS967 (Figure 2A) and TTX (Figure 2B), I_NaL_ current densities at 50% of APD_90_ were significantly lower in the presence of nisoldipine (Figure 2C). Similarly, current integrals were smaller in nisoldipine; however, this difference was only marginally significant (*p* = 0.1) in the case of TTX (Figure 2D).

There are two possible explanations for this behavior. The first option is that GS967 (and TTX as well) might also suppress I_Ca_ at the applied concentration. In this case, when GS967 is used without L-type calcium channel blockade, the GS967-sensitive current would be contaminated with a small fraction of I_Ca_. The second option is that I_NaL_ could be modulated by changes in intracellular Ca^2+^ concentration. In this case, I_NaL_ becomes smaller, as [Ca^2+^]_i_ is reduced by nisoldipine pretreatment. To test the first possibility, we studied the effect of GS967 on I_Ca_ under conventional voltage-clamp conditions.

### 2.2. Effect of GS967 on I_Ca_ under Conventional Voltage Clamp Conditions

**Figure 3:**
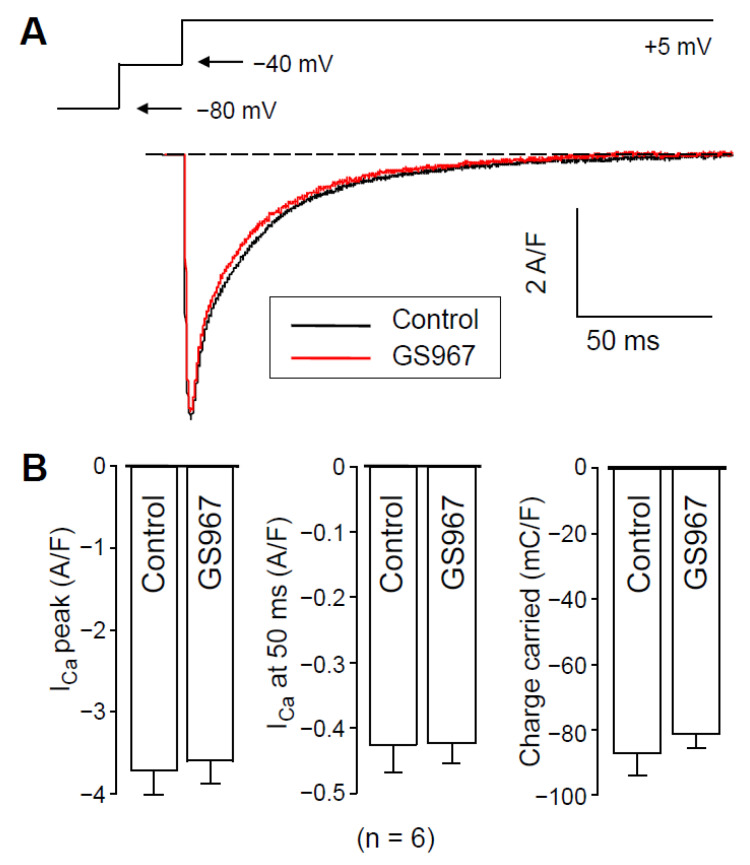
Effects of 1 µM GS967 on I_Ca_ under conventional voltage-clamp conditions using test pulses of 200 ms duration clamped to +5 mV from the holding potential of −80 mV following a 15 ms duration prepulse to −40 mV. (**A**) Representative superimposed analog I_Ca_ records. (**B**) Average I_Ca_ densities measured as peak currents, or 50 ms after beginning the pulse and I_Ca_ integrals obtained in six myocytes. Columns and bars represent mean ± SEM values.

Since action potential clamp experiments raised the possibility that GS967 might interfere with I_Ca_, we investigated this possibility using conventional voltage-clamp experiments. As shown in Figure 3A, GS967 caused no change in the profile of I_Ca_. Neither peak I_Ca_, nor its density measured at 50 ms after the beginning of the pulse, was altered by 5 min perfusion with 1 µM GS967 (Figure 3B). Similarly, no change was observed in the current integral measured before and after the application of GS967 (Figure 3B). This result excludes the contamination of the GS967-sensitive current (I_NaL_) with I_Ca_ and supports the selective action of GS967 on I_NaL_. Therefore, nisoldipine pretreatment most likely reduces I_NaL_ (Figure 2) because of the [Ca^2+^]_i_-dependent behavior of I_NaL_, as Hegyi et al. also reported previously in rabbit myocytes [18].

### 2.3. Effects of GS967 on I_NaL_ in the Presence of Intracellular BAPTA

**Figure 4:**
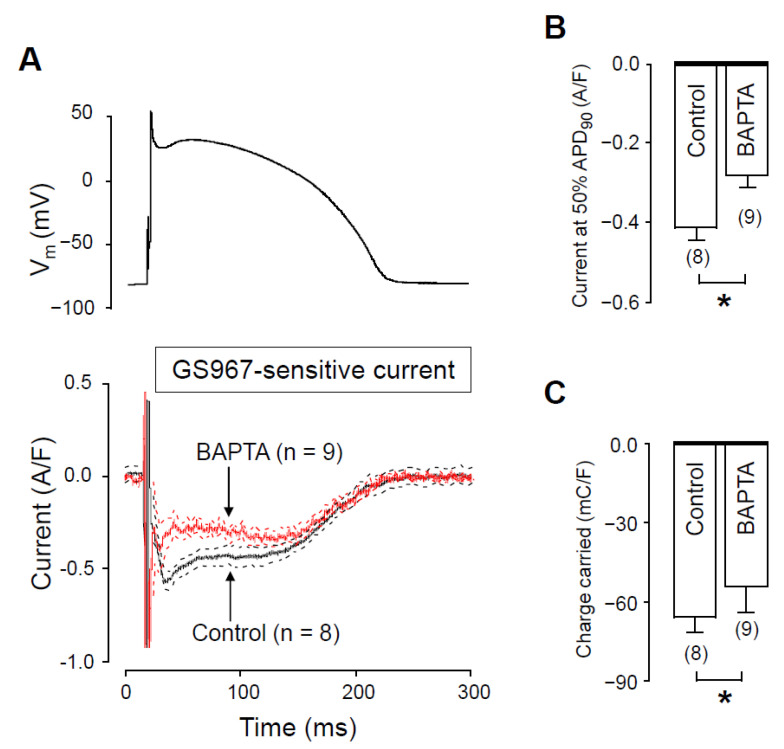
Effects of GS967 on I_NaL_ under action potential voltage clamp conditions in control and in the presence of 10 mM BAPTA, added to the pipette solution. (**A**) The command AP (above) and GS967-sensitive current profiles (below) were obtained in the presence or absence of BAPTA. The records represent average data from eight and nine myocytes, respectively, and the dashed lines denote SEM values. (**B**,**C**) Average I_NaL_ densities, measured at 50% of APD_90_ (**B**) and current integrals (**C**) obtained in the absence and presence of BAPTA. Columns and bars are mean ± SEM, numbers in parentheses indicate the number of myocytes studied, asterisks denote significant differences between the BAPTA-treated and control groups.

To further support the role of [Ca^2+^]_i_ in the regulation of I_NaL_, we reduced cytosolic Ca^2+^ by applying 10 mM BAPTA in the pipette solution. The current profiles obtained with and without BAPTA are demonstrated in Figure 4A. In these experiments, measurements started 10 min after rupturing the seal to let the Ca^2+^ chelator BAPTA equilibrate between the pipette solution and the intracellular space. In the presence of intracellular BAPTA, both I_NaL_ density at 50% of APD_90_ and the current integral were significantly lower than obtained under control conditions. I_NaL_ densities at 50% of APD_90_ were −0.30 ± 0.03 A/F vs. −0.42 ± 0.03 A/F (Figure 4B) whereas I_NaL_ integrals were −46.7 ± 5.2 mC/F vs. −68 ± 5 mC/F (Figure 4C), in the presence versus in the absence of BAPTA, respectively.

### 2.4. Effects of Nisoldipine and BAPTA-AM on Unloaded Cell Shortening

**Figure 5:**
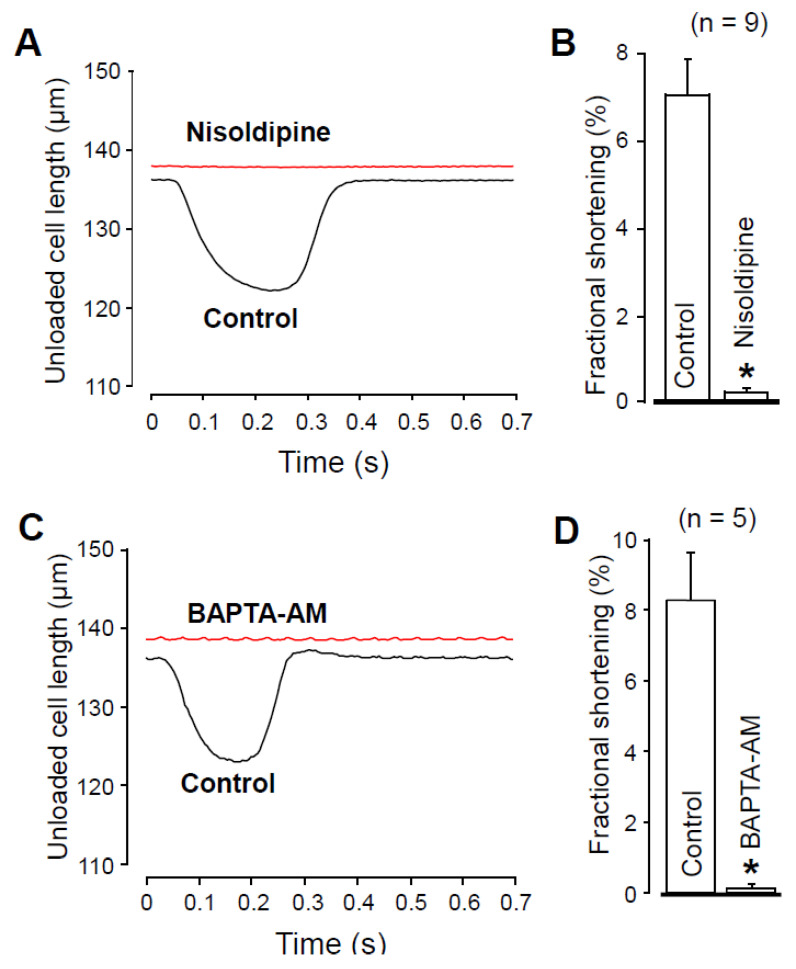
Recording of unloaded cell shortening in the absence and presence of 1 µM nisoldipine (**A**,**B**) and after superfusion with 5 µM BAPTA-AM for 30 min (**C**,**D**). (**A**,**C**) Representative superimposed records of unloaded cell length. Downward deflection indicates cell shortening. (**B**,**D**) Average results of fractional cell shortening. Columns and bars are mean ± SEM values, numbers in parentheses indicate the number of myocytes studied, asterisks denote significant differences in control and post-drug data.

It would have been reasonable to compare [Ca^2+^]_i_ in the absence and presence of nisoldipine. However, it is technically difficult because nisoldipine is rapidly degraded by the UV light required for the fluorescent measurement of [Ca^2+^]_i_. Therefore, to indirectly demonstrate the effect of 1 µM nisoldipine on [Ca^2+^]_i_, we recorded unloaded cell shortening. Figure 5 clearly shows that either 1 µM nisoldipine treatment (Figure 5A,B) or 30 min pretreatment with 5 µM BAPTA-AM (Figure 5C,D) decreased the fractional shortening practically to zero. These results suggest a diminished [Ca^2+^]_i_ transient both in the presence of nisoldipine and after 30 min BAPTA-AM pretreatment.

### 2.5. The Role of CaMKII in Regulation of I_NaL_

**Figure 6:**
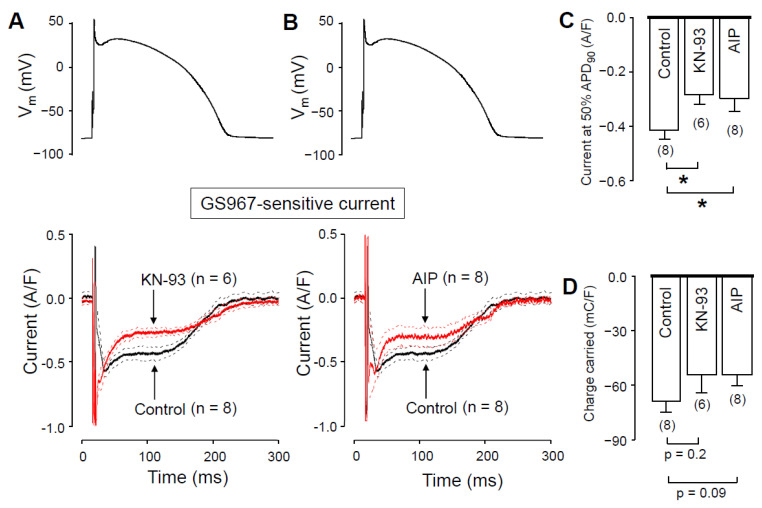
Effects of GS967 on I_NaL_ under action potential voltage-clamp conditions in control and in the presence of the CaMKII inhibitor KN-93 and AIP added to the pipette solution. (**A**,**B**) Command APs (above) and GS967-sensitive current profiles (below) were obtained in the presence or absence of 1 µM KN-93 (**A**) and 0.5 µM AIP (**B**). The records represent average data, and the dashed lines denote SEM values. (**C**,**D**) Average I_NaL_ densities, measured at 50% of APD_90_ (**C**), and current integrals (**D**) obtained in control and in the presence of KN-93 and AIP. Columns and bars are mean ± SEM, numbers in parentheses indicate the number of myocytes studied, asterisks denote significant differences between the KN-93 or AIP and control groups.

We hypothesized that the Ca^2+^-dependent augmentation of I_NaL_ is mediated by CaMKII. To test this hypothesis, we measured I_NaL_ in the presence of CaMKII inhibitors. In these experiments, the pipette solution contained either 1 µM KN-93 or 0.5 µM autocamtide-2-related inhibitor peptide (AIP), as selective inhibitors of CaMKII. We started the recording 10 min after establishing the whole cell configuration, to let KN-93 or AIP equilibrate inside the myocyte. I_NaL_ densities at 50% of APD_90_ were significantly smaller with both KN-93 (Figure 6A) and AIP (Figure 6B) than under control conditions (Figure 6C). I_NaL_ integrals were also smaller with KN-93 and AIP (Figure 6D), however, these differences did not reach the level of statistical significance (*p* = 0.2, and *p* = 0.09, respectively). Upon comparing our results obtained with KN-93, AIP, nisoldipine and BAPTA, neither the current densities measured at 50% of APD_90_ (−0.28 ± 0.04, −0.30 ± 0.05, −0.29 ± 0.04 and −0.30 ± 0.03 A/F, respectively) nor the respective current integrals (−54.6 ± 9.9, 53.4 ± 6.4, −48.2 ± 3.6 and −46.7 ± 5.2 mC/F, respectively) were significantly different among these four groups. These data show that the reduction of [Ca^2+^]_i_ with either nisoldipine or BAPTA decreases I_NaL_ just like CaMKII inhibition with KN-93 or AIP does. Our results also suggest that in the presence of nisoldipine or BAPTA, the lower [Ca^2+^]_i_ can reduce CaMKII activity so much that I_NaL_ will become significantly smaller than with normal intracellular calcium homeostasis.

### 2.6. Effect of GS967 on Action Potential Morphology

**Figure 7:**
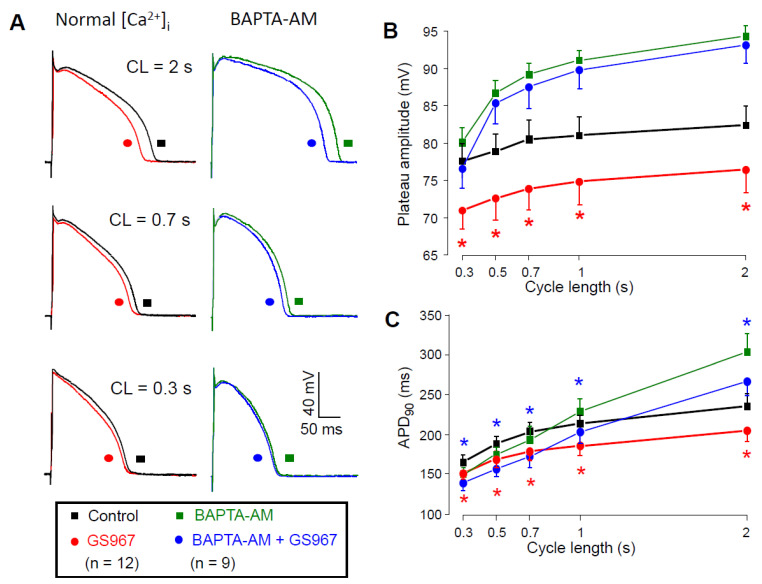
Effects of GS967 on AP configuration. (**A**) Superimposed AP pairs recorded in control and after superfusion with 1 µM GS967 at various pacing cycle lengths. These experiments were performed either under conditions of normal Ca^2+^ cycling (left side), or after pretreatment with 5 µM BAPTA-AM for 30 min (right side). (**B**,**C**) Effects of GS967 on plateau amplitude (**B**) and AP duration (**C**), as a function of the pacing cycle length. Symbols and bars are mean ± SEM, numbers in parentheses indicate the number of myocytes studied, asterisks denote significant differences between data obtained before and after the application of 1 µM GS967. Accordingly, red asterisks indicate significant differences in control (i.e., GS967 versus control), while blue asterisks indicate significant differences obtained in the presence of BAPTA-AM (i.e., GS967 + BAPTA-AM versus BAPTA-AM).

The Ca^2+^-dependent behavior of the GS967-sensitive current could also be demonstrated under current clamp conditions when APs were elicited by electrical stimulation; 1 µM GS967 significantly shortened the AP duration (measured at 90% repolarization) in a reverse rate-dependent manner and significantly decreased the amplitude of the plateau phase (defined as the difference between the plateau potential measured at 50% of APD_90_ and the resting membrane potential) as presented in Figure 7A. The GS967-induced plateau depression was significantly smaller when we pretreated the cells with 5 µM BAPTA-AM for 30 min (Figure 7B), while the magnitude of the APD shortening effect of GS967 remained similar after loading the cells with the Ca^2+^-chelator (Figure 7C). Application of BAPTA-AM increased APD_90_ and shifted the plateau phase towards more positive potentials likely due to reduction of the Ca^2+^-dependent inactivation of I_Ca_. The effect of BAPTA-AM depended on the pacing cycle length: while BAPTA-AM significantly prolonged APD at 2 s cycle length, no significant changes were observed at shorter ones (Figure 7C, black symbols vs. green symbols).

Based on our results (Figure 4), I_NaL_ becomes smaller as intracellular BAPTA chelates [Ca^2+^]_i_. Therefore, in the presence of BAPTA-AM, when a reduced I_NaL_ is inhibited with GS967, the GS967-induced APD shortening effect is expected to be smaller compared to the condition when the calcium homeostasis is undisturbed. On the contrary, the APD shortening effect of GS967 was similar with or without BAPTA-AM pretreatment. This similarity can only partially be explained by the fact that the BAPTA-AM-induced lengthening of APD developed only at longer cycle lengths (Figure 7C). It is also known that many ion channel modulator drugs change APD proportionally to the baseline APD value [35,36,37]. Therefore, our results on the GS967-induced changes in APD in the presence of BAPTA-AM likely represent the sum of three independent effects: (1) the BAPTA-AM induced APD lengthening effect at longer cycle lengths; (2) the expected reduction of the GS967-induced APD shortening effect because of the smaller I_NaL_ and finally (3) the intrinsic dependency of APD changes on baseline APD. These three effects seemed to compensate each other, causing similar GS967-induced APD shortening in the presence of BAPTA-AM throughout the investigated cycle lengths.

Our current clamp results indirectly indicate the [Ca^2+^]_i_-dependency of I_NaL_ because the GS967-induced plateau depression appeared only in cells with normal calcium homeostasis and not in the presence of BAPTA-AM.

## 3. Discussion

Our key finding is that under physiological conditions changes in [Ca^2+^]_i_ modulate I_NaL_ in canine ventricular cells. Whenever [Ca^2+^]_i_ was reduced—either by applying 10 mM intracellular BAPTA or by perfusing the cells with 1 µM nisoldipine—the density and the integral of I_NaL_ significantly decreased. The Ca^2+^-dependency of I_NaL_ was evident under both action potential voltage clamp and current clamp conditions. Furthermore, this Ca^2+^-dependent modulation of I_NaL_ was likely due to the contribution of CaMKII in regulating the current because inhibition of CaMKII with KN-93 or AIP decreased the density of the current to a similar level observed with nisoldipine or BAPTA. The CaMKII-related stimulation of I_NaL_ observed by us in healthy canine ventricular cells was like those reported in rabbit [19,20,21], murine [21,22,23], canine [14,38] and human [14] myocytes under various pathological conditions, such as heart failure, cardiac hypertrophy, ischemia or hypoxia.

Both EF hand (target of Ca^2+^-binding), as well IQ (target of CaM binding) motifs are present in the Na_v_1.5 polypeptide chain. Wingo et al. showed that Ca^2+^ itself may regulate sodium channels [39], while other authors suggest that it is the Ca^2+^/CaM complex that regulates voltage-gated sodium channels [40,41]. There is a general consensus, however, that the steady-state inactivation curve of Na^+^ channels is shifted toward more positive voltages by higher [Ca^2+^]_i_ [42]. Nevertheless, even a small shift in the steady-state inactivation curve might be enough to create a larger I_NaL_ in cardiac cells with normal calcium homeostasis, compared with the conditions when nisoldipine, BAPTA, KN-93, or AIP is applied.

Our results indicate indirectly that in paced canine ventricular cardiomyocytes with intact calcium homeostasis—just like in the physiologically active, working ventricle—CaMKII is partially activated. This is indicated by the marked reduction of I_NaL_ in response to decreasing [Ca^2+^]_i_ or inhibition of CaMKII. Previously, only Maltsev and colleagues investigated the effect of [Ca^2+^]_i_ on I_NaL_ in canine myocytes, but they only compared conditions of zero (chelated with EGTA or BAPTA) and high (1 µM) levels of [Ca^2+^]_i_ [38] at room temperature. Under these two conditions, CaMKII was most likely either completely inactive or fully activated, respectively. Even though our data represent a condition that is much closer to in vivo physiology than what Maltsev et al. [38] carried out, we did not study I_NaL_ when CaMKII is maximally activated at high [Ca^2+^]_i_ levels, so we could not estimate the total CaMKII-dependent fraction of I_NaL_.

Erickson et al. [43] also concluded that CaMKII is partially active in paced adult rabbit cardiomyocytes. They utilized the FRET-based Camui sensor to examine CaMKII activation under various conditions. In their study, the addition of Ca^2+^/CaM significantly increased both CaMKII and Camui activity in lysates of HEK cells expressing the Camui construct. Furthermore, the Camui sensor detected increasing CaMKII activation as either the pacing frequency or the bath Ca^2+^ concentration was increased in primarily isolated rabbit cardiomyocytes. Both in HEK cells and in adult cardiomyocytes, the addition of 1 μM KN-93 blocked the FRET change associated with activation of Camui. This suggests that KN-93 prevents CaMKII activation resulting from the physiological excitation-contraction coupling. This is in line with our results, where blocking CaMKII activation either by KN-93 or by AIP resulted in a smaller I_NaL_ than under control conditions.

In lysates of adult rabbit myocytes, Wood et al. also showed that CaMKII-dependent phosphorylation of CaMKII (autophosphorylation), ryanodine receptor, and phospholamban were significantly higher in paced cells than in unstimulated myocytes [44]. This also suggests a higher CaMKII activity in physiologically activated (paced) myocytes compared to completely quiescent cells. Similarly, in another study performed also on rabbit ventricular cells, Hegyi et al. attributed largely half of the basal I_NaL_ to CaMKII activity [18].

In contrast with the aforementioned results in rabbits [18,44] and with our present data in canine myocytes, Fu et al. reported that the baseline I_NaL_ density was not affected by the reduction of [Ca^2+^]_i_ or inhibition of CaMKII in rabbit ventricular cells under control, normoxic conditions, but only in hypoxic myocytes [19]. The most likely reason for this discrepancy is that both in the experiments of Hegyi et al. [18] and in our own measurements, the calcium homeostasis of the cardiomyocytes was physiologically intact. Fu and colleagues [19], however, used 10 mM EGTA in the pipette solution to chelate intracellular calcium and 10 µM nisoldipine to block the L-type calcium channels for I_NaL_ recordings. These interventions likely already significantly reduced, or completely switched off the [Ca^2+^]_i_-dependent regulation of I_NaL_, therefore, neither KN-93 nor BAPTA-AM could further reduce I_NaL_ under normoxic conditions. Fu and colleagues also showed that hypoxia increased diastolic [Ca^2+^]_i_ that leads to CaMKII activation. Furthermore, Lu et al. found that hypoxia might also stimulate CaMKII directly [45]. Therefore, CaMKII activity increases under hypoxic conditions, stimulating I_NaL_ in turn. This is the likely reason why KN-93 and BAPTA-AM reduced the *hypoxia-mediated increase* in I_NaL_.

While it is generally believed that KN-93 binds to CaMKII, thus preventing kinase activation by competing with Ca^2+^/CaM, recent data suggest that KN-93 binds directly to Ca^2+^/CaM and not to CaMKII [46]. Although the mode of action presented by Wong et al. [46] still regards KN-93 as a functional CaMKII inhibitor, the ubiquity of Ca^2+^/CaM regulation prompts the question of whether the KN-93-based observations (like data presented in our study) could partly or fully be explained by a Ca^2+^/CaM-dependent, but CaMKII-independent inhibition. To address this issue, besides KN-93 we also used AIP for CaMKII inhibition. As I_Na,L_ densities obtained with KN-93 and AIP (Figure 6) were both smaller than under control conditions and did not differ from each other, we conclude that inhibiting either CaM- or CaMKII action both decrease I_Na,L_ in a similar fashion.

Based on our findings, we also emphasize that L-type calcium channel blockers do not only inhibit the L-type Ca^2+^ current itself but as a result, [Ca^2+^]_i_ is also decreased. Therefore, these inhibitors interfere with all Ca^2+^-dependent processes, such as calmodulin-, and CaMKII-mediated actions. Because blockade of L-type Ca^2+^ channels (for example, with nisoldipine) are often used in basic research to separate drug actions targeting cardiac L-type Ca^2+^ current, it is important to keep these Ca^2+^-dependent processes in mind. Therefore, whenever L-type Ca^2+^ channel blockers are used, reduction of all Ca^2+^-, CaM-, and CaMKII-dependent processes need to be considered during the interpretation of the obtained results.

In conclusion, our results demonstrate that in canine ventricular cells [Ca^2+^]_i_ modulates I_NaL_ under physiological conditions, a process likely mediated by CaMKII.

## 4. Materials and Methods

### 4.1. Animals

Adult mongrel dogs of either sex were anesthetized with i.m. injections of 10 mg/kg ketamine hydrochloride (Calypsol, Richter Gedeon, Hungary) + 1 mg/kg xylazine hydrochloride (Sedaxylan, Eurovet Animal Health BV, The Netherlands) according to a protocol approved by the local Animal Care Committees (license N°: 2/2020/DEMáB at University of Debrecen; and I-74-15-2017, I-74-24-2017 at University of Szeged) and by the Department of Animal Health and Food Control of the Ministry of Agriculture and Rural Development (XIII/3330/2017 and XIII/3331/2017). All animal procedures conform to the guidelines from Directive 2010/63/EU of the European Parliament on the protection of animals used for scientific purposes and the Guide for the Care and Use of Laboratory Animals (USA NIH publication NO 85-23, revised 1996).

### 4.2. Isolation of Cardiomyocytes

Single canine myocytes were obtained by enzymatic dispersion using the segment perfusion technique, as previously described [47]. A wedge-shaped section of the ventricular wall supplied by the left anterior descending coronary artery (LAD) was cannulated, dissected and perfused with a nominally Ca^2+^-free Joklik solution (Minimum Essential Medium Eagle, Joklik Modification, Sigma-Aldrich Co. St. Louis, MO, USA) for a period of 5 min. After this, the tissue was perfused with Joklik solution supplemented with 1 mg/mL collagenase (Type II, Worthington Biochemical Co., Lakewood, NJ, USA; representing a final activity of 224 U/mL) and 0.2% bovine serum albumin (Fraction V., Sigma) containing 50 µM Ca^2+^ for 30 min. Finally, the normal external Ca^2+^ concentration was gradually restored, and the cells were stored at 15 °C in Minimum Essential Medium Eagle not more than 36 h until use. The chemicals used in the experiments were obtained from Sigma-Aldrich Co. (St. Louis, MO, USA).

### 4.3. Electrophysiology

Cells were placed in a plexiglass chamber under an inverted microscope allowing for continuous superfusion with a modified Tyrode solution by gravity flow at a rate of 1–2 mL/min. The modified Tyrode solution contained (in mM): NaCl 121, KCl 4, CaCl_2_ 1.3, MgCl_2_ 1, HEPES 10, NaHCO_3_ 25, glucose 10 at pH = 7.35, which was supplemented according to the actual experimental design. The osmolarity of this solution was adjusted to 300 ± 3 mOsm with the addition of NaCl or water as necessary. In all experiments, the bath temperature was set to 37 °C using a temperature controller (Cell MicroControls, Norfolk, VA, USA). Electrical signals were amplified and recorded using a MultiClamp 700A or 700B amplifier (Molecular Devices, Sunnyvale, CA, USA) under the control of a pClamp 10 software (Molecular Devices) following analog-digital conversion performed by a Digidata 1332A or 1440A converter (Molecular Devices). Microelectrodes were manufactured from borosilicate glass with a P-2000 micropipette puller (Sutter Instruments, Novato, CA, USA) and had tip resistances of 2–3 MΩ when filled with pipette solution. Transmembrane currents were recorded in whole-cell voltage clamp mode. The series resistance was typically 4-8 MΩ, and the measurement was discarded if it changed substantially during the experiment.

### 4.4. Action Potential Voltage Clamp

Action potential voltage clamp experiments were performed according to the methods described [48,49]. A previously recorded midmyocardial canine ventricular AP was applied as a command signal and the current traces were recorded continuously in modified Tyrode solution before and after 5 min superfusion with the Na^+^ channel inhibitor applied. The drug-sensitive current was obtained by subtracting the post-drug trace from the pre-drug trace. These measurements were performed either in the presence or absence of 1 µM nisoldipine added to the Tyrode solution. The pipette solution contained (in mM): K-aspartate 120, KCl 30, MgATP 3, HEPES 10, Na_2_-phosphocreatine 3, EGTA 0.01, cAMP 0.002, KOH 10 at pH = 7.3 with an osmolarity of 285 mOsm. When the effect of CaMKII inhibition on I_NaL_ was measured, 1 µM KN-93 or 0.5 µM AIP was added to the pipette solution allowing diffusion into the cell after disrupting the membrane. The amplitude of the dissected current was evaluated at 50% of APD_90_. When determining the current integral, the initial 20 ms after the AP upstroke was excluded from evaluation in order to eliminate the contribution of the fast I_Na_ component. In each experiment, 20 consecutive current traces were averaged and analyzed in order to reduce the noise and the trace-to-trace fluctuations of action potential configuration. Ion currents were normalized to cell capacitance, determined in each cell by applying hyperpolarizations from +10 to −10 mV for 15 ms.

### 4.5. Conventional Voltage Clamp

In order to exclude any contamination of the GS967-sensitive current with I_Ca_, conventional voltage clamp experiments, using rectangular command pulses, were performed to study the effects of GS967 on I_Ca_ at stable test potentials. The bath solution was modified Tyrode solution supplemented with 10 µM TTX, 1 µM E-4031, 50 µM chromanol 293B and 3 mM 4-aminopyridine to suppress Na^+^ and K^+^ currents. The pipette solution contained (in mM): K-aspartate 120, KCl 30, MgATP 3, HEPES 10, Na_2_-phosphocreatine 3, EGTA 0.01, cAMP 0.002, KOH 10 at pH = 7.3. Test pulses to +5 mV, lasting for 200 ms, arose from the holding potential of −80 mV, while a prepulse to −40 mV for 15 ms was interposed between the holding potential and the test pulse to inactivate the remaining Na^+^ current. In this arrangement, the current integral contained the total amount of current carried by I_Ca_ from the beginning to the end of the test pulse.

### 4.6. Recording of Action Potentials

Transmembrane potentials were recorded using 3 M KCl filled sharp glass microelectrodes having tip resistance between 20 and 40 MΩ as reported previously [50]. These electrodes were connected to the input of Multiclamp 700A or 700B amplifier (Molecular Devices, Sunnyvale, CA, USA). The cells were paced through the recording electrode at a cycle length of 1 s using 1–2 ms wide rectangular current pulses having suprathreshold amplitudes. Following equilibration at 1 s cycle length, the cycle length was sequentially varied between 0.3 and 2 s. At each cycle length, the 50 APs were recorded, and the cycle length was then changed. Under these conditions, a quasi-steady state rate-dependence could rapidly be obtained. Action potentials were digitized at 200 kHz using a Digidata 1332A or 1440A converter (Molecular Devices) and stored for later analysis.

### 4.7. Recording of Unloaded Cell Shortening

Unloaded cell shortening of field-stimulated myocytes was measured using an edge detector system (VED-105, Crescent Electronics, Sandy, Utah, USA) at a sampling rate of 240 Hz. The analog signal was amplified (DC amplifier, Főnixcomp Ltd., Debrecen, Hungary), digitized (Digidata 1440A, Molecular Devices) and recorded with pClamp 10 software (Molecular Devices). Cell length was calculated after calibrating the edge detector system with a hemocytometer, and fractional shortening was expressed as a percent of the initial diastolic cell length. In each cell, 10 consecutive curves were obtained before and after the application of 1 µM nisoldipine, and before and after exposure of the cells to 5 µM BAPTA-AM for 30 min. These data were averaged during offline analysis [51].

### 4.8. Statistics

Results are expressed as mean ± SEM values, n denotes the number of myocytes studied. Statistical significance of differences was evaluated using one-way ANOVA followed by a Student’s *t*-test for paired or unpaired data as pertinent. Differences were considered significant when *p* was less than 0.05.

## 5. Conclusions

- I_NaL_ depends on the magnitude of [Ca^2+^]_i_ in canine ventricular cells.
- The [Ca^2+^]_i_-dependence of I_NaL_ is mediated by the Ca^2+^-dependent activation of CaMKII.
- I_NaL_ is augmented by the baseline CaMKII activity.

## Data Availability

Data is contained within the article.
